# Integrative Oncology Education: An Emerging Competency for Oncology Providers

**DOI:** 10.3390/curroncol28010084

**Published:** 2021-02-10

**Authors:** Safiya Karim, Rita Benn, Linda E. Carlson, Judith Fouladbakhsh, Heather Greenlee, Rick Harris, N. Lynn Henry, Shruti Jolly, Sabrina Mayhew, Lisa Spratke, Eleanor M. Walker, Bradley Zebrack, Suzanna M. Zick

**Affiliations:** 1 Department of Oncology, Tom Baker Cancer Centre, University of Calgary, Calgary, AB T2N 1N4, Canada; lcarlso@ucalgary.ca; 2 Department of Family Medicine, University of Michigan, Ann Arbor, MI 48109, USA; ritabenn@umich.edu (R.B.); szick@med.umich.edu (S.M.Z.); 3 School of Nursing, Oakland University, Rochester, MI 48309, USA; dr.judi129@gmail.com; 4 Public Health Sciences & Clinical Research Divisions, Fred Hutchison Cancer Research Centre, Seattle, WA 98109, USA; hgreenlee@fredhutch.org; 5 Department of Medicine, University of Washington School of Medicine, Seattle, WA 98195, USA; 6 Seattle Cancer Care Alliance, Seattle, WA 98109, USA; 7 Department of Anesthesiology, University of Michigan, Ann Arbor, MI 48109, USA; reharris@med.umich.edu; 8 Department of Internal Medicine, University of Michigan, Ann Arbor, MI 48109, USA; norahh@med.umich.edu; 9 Department of Radiation Oncology, University of Michigan, Ann Arbor, MI 48109, USA; shrutij@med.umich.edu; 10 College of Nursing, Wayne State University, Detroit, MI 48202, USA; sabrinamayhew44@gmail.com; 11 Division of Gynecologic Oncology, University of Michigan, Ann Arbor, MI 48109, USA; lisaspra@med.umich.edu; 12 Department of Radiation Oncology, Henry Ford Hospital, Detroit, MI 48202, USA; ewalker1@hfhs.org; 13 School of Social Work, University of Michigan, Ann Arbor, MI 48109, USA; zebrack@umich.edu; 14 Department of Nutritional Sciences, University of Michigan, Ann Arbor, MI 48109, USA

**Keywords:** integrative oncology, oncology education, integrative medicine, supportive cancer care, complementary medicine

## Abstract

A growing number of cancer patients use complementary and alternative therapies during and after conventional cancer treatment. Patients are often reluctant to discuss these therapies with their oncologist, and oncologists may have limited knowledge and confidence on how to advise patients on the appropriate use. Integrative oncology is a patient-centered, evidence-informed field that utilizes mind–body practices, lifestyle modifications and/or natural products interwoven with conventional cancer treatment. It prioritizes safety and best available evidence to offer appropriate interventions alongside conventional care. There are few opportunities for oncologists to learn about integrative oncology. In this commentary, we highlight the Integrative Oncology Scholars (IOS) program as a means to increase competency in this growing field. We provide an overview of several integrative oncology modalities that are taught through this program, including lifestyle modifications, physical activity, and mind–body interventions. We conclude that as more evidence is generated in this field, it will be essential that oncology healthcare providers are aware of the prevalent use of these modalities by their patients and cancer centers include Integrative Oncology trained physicians and other healthcare professionals in their team to discuss and recommend evidence-based integrative oncology therapies alongside conventional cancer treatments to their patients.

## 1. Introduction

Many cancer patients and survivors use complementary and alternative therapies during and after cancer treatment. A recent meta-analysis of 32 surveys of cancer patients in North America found a point prevalence of 46% (95% CI 35–56%) of complementary medicine use and showed that rates of use have been growing steadily in the past few decades [1]. Patients cite a wide variety of reasons to pursue complementary therapies, including belief in their effectiveness, having control over their treatments, and finding hope [2]. However, despite the growing use of complementary therapies, there is a lack of communication about such use amongst both patients and oncology providers. A recent survey conducted by the American Society of Clinical Oncology (ASCO) found that less than half of the oncologists surveyed discussed the use of complementary and alternative therapies with their patients and only a minority of the conversations were initiated by the oncologist [3]. Surveys have found that a common reason for non-disclosure of complementary and alternative medicine use among patients was because they were never asked by their physician [4,5]. Many clinicians are reluctant to ask patients about their use of these therapies because they do not know what to do with the information once it is collected and are not knowledgeable about the risks and benefits of complementary and integrative treatments. In addition, clinicians may not know the role of complementary therapies in symptom management or the evidence base for the efficacy of these therapies. Consequently, many patients report not receiving enough information about which therapies to use, and many choose therapies on the advice of their family and friends, rather than from a health care professional [5]. This is a significant healthcare quality concern, since the lack of education of conventional healthcare providers, and inadequate communication between patients and providers may lead to potential harmful interactions, as well as lost opportunities to recommend potentially helpful evidence-based integrative therapies during a patient’s cancer journey.

One approach to address these various concerns comes from the relatively new discipline of Integrative Oncology. Integrative Oncology, is a patient-centered, evidence-informed approach that utilizes mind–body practices, lifestyle modifications and/or natural products interwoven with conventional cancer treatment [6]. Integrative oncology prioritizes safety and best available evidence to offer appropriate interventions alongside conventional care. Several evidence-based practice guidelines have been developed by the Society for Integrative Oncology (SIO) [7,8,9] with the most recent one endorsed by ASCO [10]. In addition, SIO and ASCO recently announced that they will collaborate to develop a series of five new evidence-based guidelines for integrative therapies in oncology care [11]. As more evidence becomes available, including the publication of these joint guidelines, it is essential that oncology providers be equipped with the knowledge, skills and abilities to discuss various complementary and integrative therapies with their patients. Until recently, however, no oncology specific training has been available to oncology providers wishing to broaden their knowledge.

Dr. Karim, the first author and a trained Medical Oncologist recently had the opportunity to participate in the Integrative Oncology Scholars (IOS) Program at the University of Michigan. This year long program is supported through a grant from the National Cancer Institute, and aims to train 100 integrative oncology (IO) leaders and to facilitate partnerships with IO leaders and complementary practitioners within their communities [12]. In the 2019–2020 cohort, Dr. Karim was joined by 24 multi-disciplinary oncology providers including oncologists, physician assistants, nurses, social workers, and pharmacists who were actively engaged in clinical oncology practice. The course involved three in-person sessions at the University of Michigan and completion of several eLearning activities prior to each of these sessions. The topics included a review of several cancer-related symptoms where integrative oncology therapies may have a role (i.e., fatigue, sleep, sexual health, pain, mood disorders), description and evidence for a variety integrative medicine modalities (diet, exercise, mind–body therapies, and natural health products), and communication skills for discussing integrative therapies with patients and complementary providers. The course also required that each participant complete a capstone (final) project that incorporated the learnings from the course. Through this training, Dr. Karim gained a better appreciation of the field of integrative oncology and increased her level of comfort in having discussions regarding integrative therapies with her patients.

Below is a brief summary of reflections and important learnings from this program:

## 2. Lifestyle Modifications

Lifestyle modifications, including diet, physical activity and sleep hygiene have been shown to have numerous benefits for people with cancer [13].

### 2.1. Diet

Many cancer patients inquire about the “best diet” for cancer and whether changes in their diet can have an impact on their cancer outcomes. Between 3–48% of cancer patients pursue “special diets” during their cancer treatment [14,15]. Unfortunately, many of these popular diets, (e.g., ketogenic) have not been extensively studied and hence lack robust scientific evidence for their use. In addition, some diets may cause nutritional deficiencies. The American Institute for Cancer Research (AICR) and the American Cancer Society (ACS) have produced evidence-based dietary guidelines for cancer [16,17]. Recommendations include maintaining a healthy body weight, eating a diet rich in fruits, vegetables, whole grains and legumes, and limiting intake of red and processed meats, alcohol, and sugar. Studies show that adherence to these guidelines is associated with a 17% reduction in cancer incidence [13] and a 6–30% decrease in cancer mortality, with the largest reductions in the incidence of breast, endometrial and colorectal cancers [13,18]. The National Cancer Institute [19] and the National Comprehensive Cancer Network [20] have also developed resources to guide patients on active treatment. Individualized guidance in conjunction with an oncology dietician, can also be beneficial.

### 2.2. Physical Activity

Sedentary behavior has been linked to the development of numerous cancers and is associated with increased cancer mortality, recurrence and treatment-related side effects [21]. Unfortunately, few cancer patients are physically active during or after treatment [22]. The ACS, the AICR and Cancer Care Ontario (CCO) recommend 150 min of moderately intense aerobic physical activity per week for adults [11,12,23]. In addition, the ACS suggests limiting sedentary behavior (i.e., sitting) [17] and CCO also suggests the addition of resistance training to aerobic activity post cancer diagnosis [23]. Physical activity during cancer treatment has been shown to have numerous benefits including improved quality of life and well-being [24], reduction in cancer associated fatigue [25], improvement in symptoms of depression and anxiety symptoms [26], lymphedema [27] and chemotherapy induced peripheral neuropathy [28]. In addition, a variety of physical activity interventions have been shown to reduce cancer specific mortality in particular for colorectal [29], breast [30] and prostate cancers [31,32]. Patients undergoing cancer treatment should undergo a pre-exercise assessment by a trained medical professional and when possible should exercise in a group or supervised setting [23].

## 3. Mind—Body Therapies

Mind-body therapies are a group of techniques that enhance the mind’s interaction with bodily function, to induce relaxation and to improve overall health and well-being [33]. These types of therapies demonstrate how physical health is connected with psychological and spiritual wellness. The most popular and commonly researched mind–body therapies include meditation, yoga, tai chi, guided imagery, and hypnosis.

### 3.1. Meditation

There are several different forms of meditation that have been studied in patients with cancer. The most studied and those with the best evidence are interventions derived from mindfulness based stress reduction (MSBR) or mindfulness based cognitive therapy (MBCT) [34,35,36,37], collectively referred to as mindfulness based interventions (MBI). Mindfulness within the context of these therapeutic interventions involves learning to pay attention to the activity of the mind in the present moment as a means to reduce exacerbation of emotional and physical distress and pain. A recent systematic review and meta-analysis of MBIs for psychosocial and physical health outcomes in cancer patients and survivors showed significant improvements in psychological distress, anxiety, depression, fear of cancer recurrence, fatigue, sleep and pain [38].

### 3.2. Yoga

Yoga is a mind–body therapy which combines physical poses (asanas) with breathing and meditation [39]. There are many different styles of yoga including hatha, Iyengar, restorative and vigorous activity types. The style of yoga should be tailored to the cancer patient or survivor based on their physical ability as well as the desired spiritual elements. A recent review of yoga in people with cancer, which included 29 RCTs, consistently found that yoga improved multiple domains of quality of life, fatigue, sleep, and psychological outcomes [40]. Yoga therapy ideally should be done under the care of a certified yoga instructor that is qualified to work with cancer patients.

### 3.3. Tai Chi and Qigong

Tai Chi and Qigong (TCQ) integrate elements of traditional Chinese medicine, martial arts conditioning, and lifestyle philosophy. These mind–body interventions incorporate elements of slow gentle movement, awareness and breath regulation in addition to intentional direction of thoughts, attention, imagery and sensation [41]. A recent systematic review and meta-analysis of TCQ for cancer-related symptoms and quality of life found statistically significant and clinically meaningful improvements in fatigue and sleep [42]. Smaller, but statistically significant effects were also observed for quality of life and depression, and preliminary evidence showed improvements in pain. While larger clinical trials and longer-follow up is required before definitive conclusions can be made regarding the role of TCQ, these interventions have promise in addressing cancer-related symptoms and improving quality of life.

### 3.4. Acupuncture and Acupressure

Acupuncture is a non-pharmacologic modality that arises from traditional Chinese medicine. It involves inserting thin needles through the skin in specific locations called acupoints to stimulate energy flow in the body. Acupressure involves stimulating the acupoints using the thumbs or fingers, without the insertion of needles. Acupuncture and acupressure have been studied for a variety of cancer-related side effects. Acupuncture has been shown to reduce aromatase inhibitor-associated joint symptoms [43,44]. Acupressure and electroacupuncture can also be considered as an addition to standard antiemetic drugs to control nausea and vomiting during chemotherapy [10]. Research has shown acupressure to be beneficial for cancer related fatigue [45,46]. Small studies have shown some benefit of acupuncture (manual or electroacupunture) for hot flashes in breast cancer patients [47] and limited evidence has shown benefit for xerostomia in head and neck cancer patients undergoing radiation [48]. Several small trials have shown that acupuncture is effective in treating chemotherapy induced peripheral neuropathy (CIPN) [49,50,51,52]. However, one study showed that electroacupuncture increased the incidence of grade 3 CIPN in breast cancer patients on taxane chemotherapy [53].

## 4. Natural Health Products

Natural health products (NHP) supplement the diet and consist of one or more of the following ingredients: vitamin, mineral, herb or botanical, or amino acid [54]. In Canada, natural health products are regulated by Health Canada under the *Natural Health Products Regulations* and require a product license and specific labeling requirements [55]. Concerns over using natural or dietary supplements while undergoing conventional cancer treatment include potential interactions in combining these treatments (herb–drug interactions) as well as using various supplements in combination with each other (herb–herb interactions) [56,57]. Additionally, in patients undergoing surgery, NHP use may impact bleeding time, anesthetic effects or cardiovascular effects [58]. Several supplements have been shown to cause harm in patients with cancer, including alpha-tocopheral and beta-carotene in head and neck patients undergoing radiation [59], and actetyl-L-carnitine in patients undergoing adjuvant breast cancer therapy [60]. On the other hand, the ASCO and SIO guidelines suggest that some natural health products may be considered. These include ginger as an addition to antiemetic drugs for control of nausea and vomiting during chemotherapy, American ginseng for cancer-related fatigue, and subcutaneous mistletoe for quality of life [7,10]. Oncologists should rely on pharmacists, integrative oncologists or reliable databases (Table 1) to assist in determining potential interactions and benefits of NHPs.

## 5. Communication with Patients and Providers

Communication with patients regarding complementary and integrative therapies, and with complementary providers is an important part of patient-centered cancer care. Several studies indicate that the reason for non-disclosure of these therapies among patients include anticipation of doctor disapproval or doctor disinterest [61]. On the other hand, when patients perceived their doctors to be respectful, open-minded, and willing to listen, they are more likely to reveal the use of complementary therapies [62]. An important component of the IOS program was to learn effective communication techniques to discuss use of complementary therapies, and to understand the patient’s motivation to engage in these therapies, which may include improving side-effects caused by medical treatment, the need for increased emotional support and humanistic care, and to improve quality of life [61]. Finally, effective communication with a patient’s complementary medicine provider is important, and failure to communicate may erode the therapeutic relationship with the patient. The framework proposed by Schiff et al. can be considered to improve effective communication [63].

## 6. Conclusions

Integrative oncology is growing field that uses the best available evidence, which incorporates safe and effective complementary therapies along conventional cancer treatment in a patient-centered approach. It is imperative that as more individuals with cancer use complementary and integrative therapies and as more research in this field emerges, oncologists should routinely ask their patients about the use of these therapies and include Integrative Oncology-trained team members who are equipped with the communication skills and competencies to guide patients in their decision making. It is important to recommend evidence-based integrative oncology therapies and separate them from those without adequate evidence to improve multiple aspects of cancer care. Evidence-based integrative therapies have the potential to improve quality of life of cancer patients and several studies have highlighted that an integrative approach is cost effective [64,65,66].

Since completing the IOS program in June 2020, Dr. Karim reports much more confidence in discussing complementary therapies with patients, and recommending evidence-based modalities for their specific concerns. We highly recommend that oncology healthcare providers build their knowledge in the field of integrative oncology, either through a formal program like the IOS program or through continuing medical education activities (Table 2).

## Figures and Tables

**Table 1:** Natural Health Product Resources.

About Herbs (Memorial Sloan Kettering Cancer Centre)	http://www.mskcc.org/aboutherbs
Natural Medicines Comprehensive Database (requires subscription)	https://naturalmedicines.therapeuticresearch.com/
Licensed Natural Health Products Database (Government of Canada)	https://www.canada.ca/en/health-canada/services/drugs-health-products/natural-non-prescription/applications-submissions/product-licensing/licensed-natural-health-products-database.html
Office of Dietary Supplements (National Institutes of Health)	https://ods.od.nih.gov/
National center for integrative and complementary health	https://www.nccih.nih.gov/
National Cancer Institute Evidence-based Physician Data Query PDQ summaries	https://www.cancer.gov/about-cancer/treatment/cam/hp

**Table 2:** Integrative Oncology Education Programs and Resources.

University of Michigan Integrative Oncology Scholars Program	https://sites.google.com/umich.edu/ioscholars/home
Memorial Sloan Kettering Cancer Centre Integrative Medicine Online Education Courses	https://www.mskcc.org/departments/division-subspecialty-medicine/integrative-medicine/programs
MD Anderson Integrative Medicine Conferences and Events	https://www.mdanderson.org/research/departments-labs-institutes/programs-centers/integrative-medicine-program/conferences-events.html
Society of Integrative Oncology Annual Conference and Webinars	https://integrativeonc.org/conference
Osher Collaborative for Integrative Medicine	https://www.oshercollaborative.org/
University of Arizona’s Introduction to Integrative Oncology Course	https://integrativemedicine.arizona.edu/online_courses/intro_oncology.html
